# High-quality host plant diets partially rescue female fecundity from a poor early start

**DOI:** 10.1098/rsos.211748

**Published:** 2022-02-23

**Authors:** Lauren A. Cirino, Patricia J. Moore, Christine W. Miller

**Affiliations:** ^1^ Entomology and Nematology Department, University of Florida, Gainesville, FL 32611, US; ^2^ Department of Entomology, University of Georgia, Athens, GA 30602, US

**Keywords:** catch-up growth, temporal, trade-offs, reproductive effort, fluctuating resources, fertility

## Abstract

Nutrition is a dynamic environmental factor and compensatory growth may help animals handle seasonal fluctuations in their diets. Yet, how the dynamic changes in nutrition affect female reproduction is understudied. We took advantage of a specialist insect herbivore, *Narnia femorata* Stål (Hemiptera: Coreidae), that feeds and reproduces on cactus across three seasons. We first examined how cactus quality can affect female reproductive success. Then, we investigated the extent to which reproductive success can be improved by a switch in diet quality at adulthood. We placed *N. femorata* juveniles onto prickly pear cactus pads with early-season (low-quality) or late-season (high-quality) fruit and tracked survivorship and development time. A subset of the females raised on low-quality diets were provided with an improved adult diet to simulate a seasonal change in diet. Adult female survival and egg production were tracked over time. All fitness-related traits were lower for females fed low-quality diets compared with females fed high-quality diets. However, when females had access to an improved adult diet, egg production was partially rescued. These findings show that a seasonal improvement in diet can enhance reproduction, but juvenile nutrition still has lasting effects that females cannot overcome.

## 1. Introduction

Poor nutrition in early life stages can have strong negative effects on fitness-related traits [1–5]. In some cases, animals can overcome poor early life diets through improvements in nutrition during adulthood [6–12]. Female reproductive traits, such as ovary size and fecundity, can often be recovered [6–9]; however, poor early nutrition can have permanent consequences for adult survival [8,13]. The studies that have teased apart the effects of nutrition at different life stages on female reproductive traits have done so through the use of artificial [8,9] or partially artificial [6] diets. While these studies have been quite valuable, the relevance of responses to poor early life conditions under natural food conditions is still unclear. Animals may have evolved adaptations to better handle nutritional changes in the foods they eat in the wild due to their evolutionary history with these foods. Thus, selection should favour recovery of all fitness-related traits from a poor early nutritional start when fed higher quality diets at adulthood. This pattern may only become apparent if fed the foods they consume in nature.

**Figure 1.**
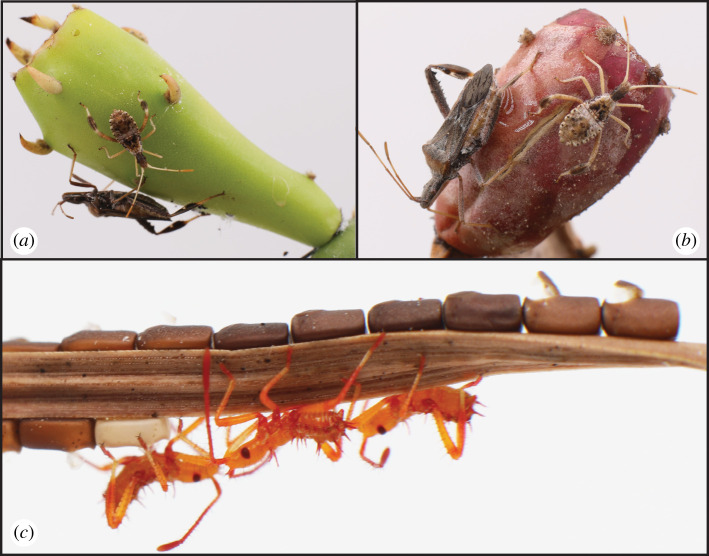
Female and 4th instar juvenile *N. femorata* on cactus pads with (*a*) early-season fruit and (*b*) late-season fruit. (*c*) Newly hatched first instar *N. femorata* offspring and the eggs from which they hatched. Photo credit: C. W. Miller.

Herbivores that feed on a single plant species throughout the year provide an excellent opportunity to investigate how natural diets (i.e. host plants) affect female reproductive traits. Some herbivores use the same plants as they flower and fruit, during which time the nutritional quality often changes dramatically [14–16]. One such species is *Narnia femorata* Stål (Hemiptera: Coreidae), the leaf-footed cactus bug (figure 1). This bug is a cactus plant specialist that lives and feeds on prickly pear cactus (*Opuntia mesacantha *ssp*. lata*) in North Central Florida [17,18]. *Narnia femorata* has a long breeding season that lasts approximately seven months in which multiple overlapping generations are produced [17]. Interestingly, *N. femorata* can develop and reproduce throughout the cactus growing season from early in the season when the cacti are just beginning to flower to the time when all cactus fruit has fully ripened and turned red [17]. Previous studies show that *N. femorata* raised on cactus pads with unripe fruit grow smaller bodies [19–21], males grow smaller testes relative to body size [20] and males raised on the season's earliest unripe fruit have smaller weapons that are less puncture-resistant [21], though we do not know the specific nutrients that these bugs require. This research highlights that the cactus fruit that appears early in the breeding season is lower quality compared with fruit that has had the chance to mature and ripen. Thus, we predicted that an early-season cactus fruit diet (i.e. unripe cactus fruit) should also have negative consequences for fitness-related traits of females.

Here, we provide an experimental study using host plant diets to test the hypothesis that females can overcome poor early life conditions through improvements in nutrition during adulthood. Alternatively, we hypothesized that poor early life nutrition will have permanent effects on female fitness regardless of nutritional improvements later in life (the silver spoon hypothesis) [22]. We examined the phenotypic effects of a lifetime of early-season (low-quality diet) and late-season fruit (high-quality diet) as well as a switch from early- to late-season fruits at adulthood. We predicted that (i) low-quality host plant diets will have negative impacts on all fitness-related traits of females relative to a high-quality diet. (ii) A switch in host plant quality at adulthood will improve all fitness-related traits because selection favours compensatory growth that avoids the fitness costs of a poor early start. We raised *N. femorata* on two different host plant quality diets: early-season and late-season cactus fruit. We switched a subset of the former treatment to late-season cactus fruit once they reached adulthood. We tracked juvenile and adult survivorship, recorded juvenile development time, and quantified long-term egg number and hatching success (fecundity).

## 2. Methods

### 2.1. Experimental design

#### 2.1.1. Prickly pear cactus phenology and *Narnia femorata* feeding ecology

The prickly pear cactus plant is composed of green flat leaves called cladodes or pads and reproductive flowers that become fruit once fertilized. In *Opuntia ficus-indica* (L.) Miller, a congener grown commercially for human and livestock consumption, cactus fruit crude fibre, protein, fat and pectin content are high early in the growing season (40–60 days from flowering) and gradually decrease as the season progresses (70–100 days from flowering) [23]. Sugar content, however, drastically increases during this time [23]. These nutrient changes over the growing season are concomitant with fruit enlargement as well as a reduction in fruit firmness [23]. Mature cactus pads are primarily composed of water and carbohydrates, although these pads also have small amounts of protein, fat and crude fibre [24].

*Narnia femorata* primarily feeds upon the fruits of the *Opuntia* cactus plant, though they also feed on the cactus pads [17,25,26]. They access the nutrients inside these plants with their long, straw-like, piercing-sucking mouthparts [27]. Since *N. femorata* feed on these cacti across the entire growing season, the seasonal changes that these plants undergo induce a variety of changes in the *N. femorata* phenotype [19–21], which we explore further in this study.

#### 2.1.2. Insect rearing

We used a mix of wild-caught (*N* = 88) and laboratory-reared (*N* = 32) *N. femorata* as parental pairs for this experiment. We collected both wild-caught parents and mature prickly pear cactus pads from Starke, Florida, USA (29.9804° N, 81.9848° W). Wild-caught parents were collected in late March to May 2018 or late in the previous breeding season (October–November 2017). We harvested cactus pads early in the growing season (March–June 2018) when the fruits on the plants were green and unripe [17,23]. We also harvested cactus pads bearing red ripe fruits late in the season (i.e. winter) of the previous year (December 2017–January 2018), planted in approximately 2.5 cm of topsoil within 32 oz deli cups enclosed with mesh lids and kept in a greenhouse (14 : 10 L/D) until the experiment began in the spring. These fruits remained red and a viable food source for the bugs. Collecting cactus pads at these two time points enabled us to provide insects with two distinct cactus fruit diets that varied in quality [23]. Cactus pad size was restricted to the size of the 32 oz deli cup containers they were planted in. When temperatures were cooler (December 2017–March 2018), cactus pads were watered up to twice a week, as needed. As temperatures warmed (April 2018–October 2018) soil moisture was checked daily, and cactus pads were watered every other day or daily, if needed.

We haphazardly created 60 parental pairs and placed each into their own 32 oz deli cup enclosed with mesh lid with a potted cactus pad bearing late-season fruit. We used a pine needle as an oviposition substrate for ease of egg transfer and because eggs of *N. femorata* have been found on fallen pine needles in the wild (LA Cirino 2015, personal observation). We separated egg clutches from the parents and kept them in their own deli cup with a cactus pad bearing late-season fruit to hatch and develop. We ensured that groups of 1st–3rd instar juveniles ranged from 5–12 individuals. This group size is common in the wild (LA Cirino 2015, personal observation) and developing in groups has a positive effect on juvenile survivorship during early life stages [21,28]. We kept parental pairs and 1st–3rd instar juveniles in incubators (Percival Scientific I-30VL; www.percivalscientific.com, 14 : 10 L/D photoperiod at 26°C), and we checked them daily for food quality (e.g. brown spots, shriveled fruit) to help ensure insect survival and the presence of 4th instar juveniles.

#### 2.1.3. Juvenile survivorship and development

*Narnia femorata* is a hemimetabolous insect that has five distinct juvenile stages of development [26]. Juveniles take about four weeks to mature from egg hatch to the penultimate (4th) instar and about another six weeks to mature from the 4th instar to adult eclosion when fed a high-quality diet and kept in constant 25°C incubators [26]. We assigned juveniles to different diets at the 4th instar because ovary development increases in size considerably at this stage of development in hemipterans [29] and was most logistically feasible due to the delicate stages of early developmental life. Additionally, *N. femorata* males grow small testes relative to their body size when fed early-season cactus fruit starting at the 4th instar compared with late-season cactus fruit-fed males [20]. We isolated 4th instar juveniles into a 32 oz deli cup with a cactus pad and fruit within 0–24 h after eclosion between 30 April and 12 June 2018. We restricted our experiment to this timeframe because the early-season cactus fruit should be of especially low quality at this time [21]. Recent findings have shown that mid-season fruit rapidly switches from a low-quality resource to a high-quality resource over a short time period [21]. We randomly assigned juveniles to either an early-season (*N* = 209) or a late-season (*N* = 206) cactus fruit diet (table 1), and they fed *ad libitum*. We transferred these individuals to a greenhouse (21–32°C, artificial light 14 : 10 L/D) until they were sexually mature adults. Female insects lived in the greenhouse between 9 May and 17 July 2018. During this time, natural light fluctuated by approximately 32 min with natural light never decreasing below 13.5 h [30]. We used greenhouse artificial fluorescent lights (Sylvania Octron/Eco 3500 K 32 W) set at a 14 : 10 L/D photoperiod to supplement natural light. The average ambient temperatures varied between 21.82°C and 28.71°C and never fluctuated more than 17.57°C within the day [31]. We checked each individually housed juvenile, along with its potted cactus pad with fruit, daily for survival, adult eclosion, and to ensure cactus pads and fruit were not deteriorating. We replaced cactus pads and fruits if there were visible signs of decay (e.g. brown spots, shriveled fruit), which was infrequent.

**Table 1.** Diets for juvenile and adult *Narnia femorata*.

juvenile diet of cactus pads with	adult diet of cactus pads with	diagram	diet quality	treatment term
early-season fruit	early-season fruit	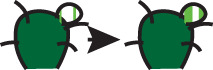	low	early season
late-season fruit	late-season fruit	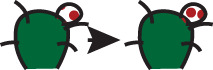	high	late season
early-season fruit	late-season fruit	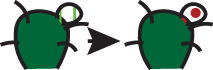	switch	switched

#### 2.1.4. Patterns of adult survivorship and fecundity

We determined sex upon adult eclosion and switched a randomly selected subsample of females from the early-season fruit diet to a late-season fruit diet within 0–24 h after adult eclosion (switched fruit *n* = 18; table 1). This change in diet quality represents a seasonal shift that *N. femorata* experiences in the wild each summer. The rest of the females that were fed early-season fruit and all females fed late-season fruit as juveniles remained on their juvenile diets as adults (early-season fruit *n* = 20, late-season fruit *n* = 27; table 1). All females in this part of the experiment came from 27 parental pairs; therefore, some females in this experiment were full siblings. However, full siblings were nearly evenly distributed across diets. We checked all adult females daily for mortality. Once females were 14 days post adult eclosion (sexual maturity in this species; [32]), we transferred them to a rearing room (14 : 10 L/D, 26°C, 60% humidity). Five lighted heat lamps (Sylvania Brooder Heat Lamp 125 W/120 V) slightly illuminated the room for 24 h a day, 4 days a week. A large opaque tarp was placed between the shelving units where our females were housed and where the heat lamps were located to reduce the amount of artificial light at night (ALAN) that reached the females in this experiment. ALAN has numerous negative effects on reproduction in animals [33–36]. However, all females, across diet treatments, received the same amount of ALAN.

We paired females with an unrelated male of similar age that was raised on a late-season fruit diet. We placed males into deli cups with females in the rearing room for 4 days along with a pine needle as an oviposition substrate. Previous work suggests that 4 days is sufficient time for long-term female fertility [37]. After the 4-day mating period, we removed the male and checked females daily from this point onward (18 days post adult eclosion) for egg laying and survival. If eggs were present, we removed each clutch and placed it into its own separate deli cup for hatching. We recorded hatching success by clutch (Y/N) after two weeks, a sufficient time for hatching [26]. We continued to check for eggs until female death or until the female had been alive for 102 days post adult eclosion. We selected this adult age because it is probably beyond typical female survival in the wild [17] and well beyond peak reproductive output of 35 days on a high-quality diet [37]. Further, females fed high-quality diets produce greater than 80% of their total eggs in the first 70 days [37]. Since some females in this study also consumed low-quality diets, they may have needed more time to accumulate resources to produce eggs, which may delay egg laying (e.g. [38]).

We cold-euthanized females that survived 102 days post adult eclosion and photographed the bodies of all experimental females, including those that died before this timepoint, next to a micro-ruler using a digital camera (Canon EOS 50D, Canon, Tokyo, Japan). We used ImageJ software (v. 1.42d) [39] to procure pronotum width measurements, a commonly used metric for body size in *N. femorata* [17,19, 40] and in Insecta (e.g. [41–43]). Since external body size is fixed at adulthood in insects, these measurements reflect the juvenile diet we provided these females rather than adult condition [44].

### 2.2. Statistical analyses

#### 2.2.1. Juvenile survivorship and development

To understand how diet affected juvenile survival (4th instar to adulthood), we ran a generalized linear model (GLM) with juvenile diet (early-season fruit *n* = 209, late-season fruit *n* = 206) as the explanatory variable and survival to adulthood (Y/N) as the response variable. We assumed a binomial distribution with a logit link function. We included both male and female insects in this juvenile survival model as sex cannot be visually determined in *N. femorata* during juvenile development.

We examined whether diet affected development time starting from the 4th instar stage of development until adult eclosion (early-season fruit *n* = 85, late-season fruit *n* = 94). We constructed a parametric proportional hazards model (time-to-event) with a lognormal distribution (due to over-dispersion) and a log link function. Diet was the explanatory variable and juvenile development time (4th instar to adult eclosion) was the response variable.

Finally, we investigated how juvenile diet affected female external body size. For this analysis, we considered all adult females used in this experiment (early-season fruit *n* = 20, late-season fruit *n* = 27, switched fruit *n* = 18). Sample sizes are lower for this analysis compared with the previous analyses because the previous analyses included all insects that were used in both this experiment and a companion experiment. We constructed a linear model with juvenile diet (early-season fruit, late-season fruit) as the explanatory variable and pronotum width (i.e. body size) as the response variable.

#### 2.2.2. Overall female reproductive success

In the following analyses, we considered all adult females including those that died before sexual maturity and thus had zero fecundity (early-season fruit *n* = 20, late-season fruit *n* = 27, switched fruit *n* = 18).

First, we constructed a GLM assuming a Poisson distribution with a log link function to investigate how diet affects egg production. Diet (early-season fruit, late-season fruit, switched fruit; table 1) was the explanatory variable and total egg number was the response variable. We then used the multcomp package in R with single-step adjusted *p*-values to perform a Tukey's *post hoc* test [45]. Next, we constructed a GLM assuming a binomial distribution with logit link function to evaluate the probability of each egg hatching. We removed all females that did not lay eggs for this analysis (early-season fruit *n* = 6, late-season fruit *n* = 26, switched fruit *n* = 13). Again, diet was included as the explanatory variable and a matrix of hatched and unhatched eggs was used as the response variable. Initially, we detected that diet affected the proportion of hatched eggs. To determine if this result was driven by three females (from the late-season fruit and switched fruit diets) that did not have any hatching success, we re-analysed the data as described above with these females removed.

#### 2.2.3. Components of female reproductive success

##### 2.2.3.1. Patterns of adult female survivorship

Next, we separately evaluated how diet affected two factors, adult survival and fecundity, and examined how these factors contributed to overall female reproductive success identified above. First, we constructed a GLM assuming a Poisson distribution with a log link function to investigate the effects of adult survivorship on reproductive success (*N* = 65). The total number of days survived was the explanatory variable and the total number of eggs laid was the response variable. Next, we compared adult survival between females from each of the three diet groups (early-season fruit *n* = 20, late-season fruit *n* = 27, switched fruit *n* = 18) by constructing three Cox proportional hazards models (early-season fruit versus late-season fruit, late-season fruit versus switched fruit and early-season fruit versus switched fruit). In each model, diet was included as the explanatory variable and survivorship (days from adult eclosion to natural death or the end of the experiment) was the response variable.

##### 2.2.3.2. Patterns of adult female fecundity

We then examined the effects of diet on the number of eggs that females produced, focusing on the females that were alive in a treatment at a given week throughout the duration of the experiment (survived to reproductive age: early-season fruit *n* = 19, late-season fruit *n* = 27, switched fruit *n* = 15; and survived to end of reproductive window: early-season fruit *n* = 7, late-season fruit *n* = 21, switched fruit *n* = 11). We constructed a zero-inflated generalized linear mixed model with a negative binomial distribution (glmmTMB package in R) [46]. Diet, week and the interaction between these two variables were included as explanatory factors, and the number of eggs laid was the response variable. Week and diet were included as the zero-inflated factors (i.e. the probability of a female producing zero eggs was assumed to vary by week and by diet). Female ID was included as a random effect for both parts of this model. This model was the most parsimonious based on the comparison of AIC scores between multiple zero-inflated GLMM models using AICtab (bbmle package in R) [47].

### 2.3. Speed of adult diet rescue

Our final goal was to begin to investigate how long it took females fed the switched fruit diet to produce their first clutch of eggs. We removed all females that did not lay eggs for this analysis (early-season fruit *n* = 6, late-season fruit *n* = 26, switched fruit *n* = 13). We constructed a parametric survival regression model (time-to-event, lognormal distribution and log link function). Diet was the explanatory variable and time (adult days) to first oviposition event was the response variable. All analyses in this manuscript were performed in R v. 4.0.5 [48].

## 3. Results

### 3.1. Juvenile survival and development

**Figure 2.**
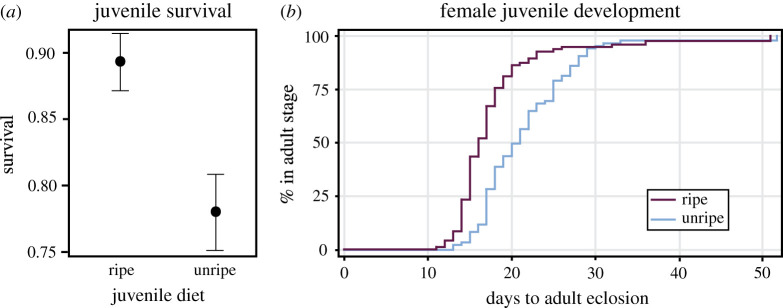
(*a*) Juveniles fed the late-season fruit were more likely to survive to adulthood than those fed an early-season fruit. Mean ± 1 s.e. bars. (*b*) Of those juveniles that survived to adulthood and were female, juveniles fed late-season fruit developed more quickly than those fed early-season fruit.

While both treatments had a relatively high probability of survival, probably a reflection of good conditions prior to treatment in the 4th instar, we found that the juveniles that were raised on late-season fruit were more likely to survive from the 4th instar to adulthood than those raised on early-season fruit (GLM, *χ*^2^ = 9.364, d.f. = 1, *p* = 0.002; figure 2*a*). Of the juvenile females that survived to adulthood, those raised on early-season fruit took 22 days, on average, to develop compared with those raised on late-season fruit that only took an average of 18 days to develop (20.2% difference) from 4th instar juveniles to adults (time-to-event, *χ*^2^ = 28.991, d.f. = 1, *p* < 0.001; figure 2*b*). Finally, females fed early-season fruit as juveniles had smaller body sizes compared with those that were fed late-season fruit (LM, *F* = 33.276, d.f. = 1, *p* < 0.001).

### 3.2. Overall female reproductive success

Females that were fed a lifetime of late-season fruit had the highest egg production of all three groups (GLM, *F* = 8.174, d.f. = 2, *p* < 0.001, electronic supplementary material, S1). Females that were switched from early- to late-season fruit at adult eclosion had lower lifetime egg production than the late-season fruit group (Tukey's test *Z* = −14.79, adjusted *p* < 0.0001), but greater egg production than the early-season fruit (Tukey's test *Z* = 15.32, adjusted *p* < 0.0001). Further, 80% of eggs hatched, on average, and did not vary based on diet for the females that laid eggs (GLM, *χ*^2^ = 2.321, d.f. = 2, *p* = 0.313, electronic supplementary material, S2). Thus, if females laid eggs, they were likely to hatch.

### 3.3. Components of female reproductive success

#### 3.3.1. Patterns of adult female survivorship

**Figure 3.**
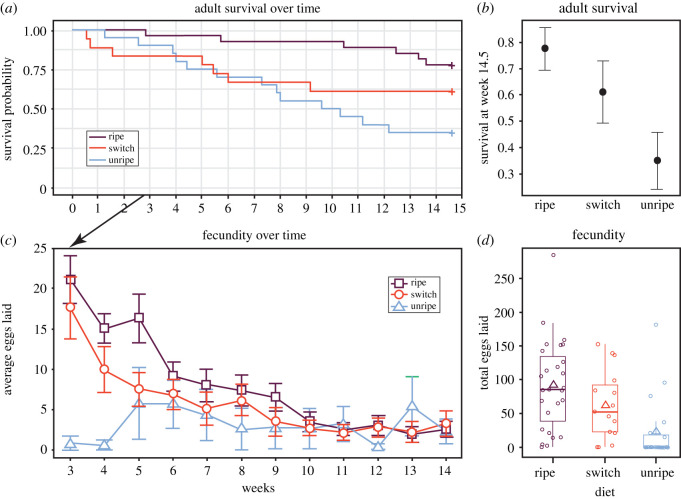
(*a*) Females fed the early-season fruit had lower adult survivorship than those females raised on the late-season fruit. Females fed the switched fruit did not differ in survivorship relative to the other groups. (*b*) Female adult survival probability at the end of the reproductive period. Mean ± 1 s.e. bars. (*c*) The arrow indicates when data collection on female fecundity began (18 days post adult eclosion). Diet and time did not together affect egg production. However, the number of eggs produced declined over time. Mean ± 1 s.e. bars. (*d*) Females fed the switched fruit were able to partially recover from a poor juvenile diet. The total number of eggs laid by each female that survived to reproductive age (open circles) are jittered over a box plot that shows the distribution, including the median (thick horizontal line) and the average (open triangles) of the total eggs laid.

We next addressed the question if the aforementioned patterns in reproductive success may be due to a difference in adult survival for females raised on different diets. We found that females that lived longer laid more eggs (GLM, *F* = 52.238, d.f. = 1, *p* < 0.001). We also found that females that were fed the early-season fruit had a lower adult survivorship than those females fed the late-season fruit (Cox proportional hazards, *χ*^2^ = 10.32, d.f. = 1, *p* = 0.001; figure 3*a*). Females that were switched from early- to late-season fruit at adult eclosion did not differ in adult survival from either of the other two diets (late-season fruit versus switched fruit: *χ*^2^ = 2.12, d.f. = 1, *p* = 0.145, early-season fruit versus switched fruit: *χ*^2^ = 1.51, d.f. = 1, *p* = 0.221; figure 3*a*). At the end of the study period (i.e. 102 days post adult eclosion), 21 of 27 (77.8%) late-season fruit-fed females, 11 of 18 (61.1%) switched fruit-fed females and 7 of 20 (35%) early-season fruit-fed females were still alive (figure 3*b*).

#### 3.3.2. Patterns of fecundity

We then examined if the overall patterns of reproductive success were related to differences in female diets. We found that diet and time affected the patterns of fecundity; however, we did not detect an interaction between these two terms (GLMM, Diet*Week: *χ*^2^ = 3.249, d.f. = 2, *p* = 0.197; Diet: *χ*^2^ = 7.374, d.f. = 2, *p* = 0.025; Week: *χ*^2^ = 91.059, d.f. = 1, *p* < 0.001; figure 3*c,d*). Egg production for all reproducing females started to decline after the first three weeks of the reproductive period (figure 3*c*). Females that were switched from early- to late-season fruit at adult eclosion produced more eggs than those females that were fed the early-season fruit but not as many eggs as the females fed the late-season fruit (figure 3*d*). Further, only 31.6% (6/19) of the surviving females fed the early-season fruit produced eggs, whereas 86.7% (13/15) of surviving females that were switched from early- to late-season fruit at adult eclosion and 96.3% (26/27) of surviving females fed the late-season fruit produced eggs.

### 3.4. Speed of adult diet rescue

**Figure 4.**
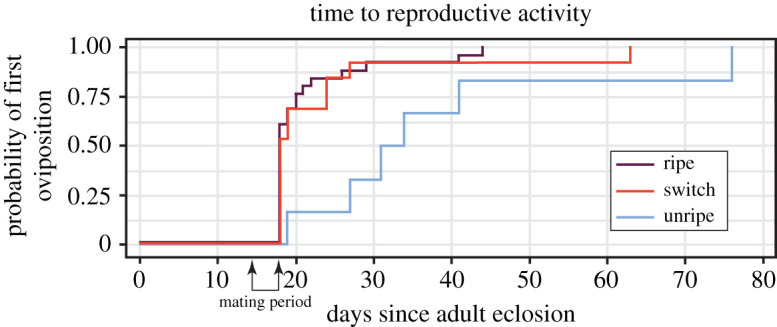
Females fed an improved adult diet (switched fruit) laid their first clutch of eggs just as quickly as females fed late-season fruit (average 23 and 21 days from adult eclosion, respectively). Females fed early-season fruit took longer (average 38 days) to oviposit than females fed the other two diets. Arrows indicate the 4 days where females were paired with a mate after adult eclosion.

We found that the amount of time it took females to oviposit for the first time did not differ between the late-season fruit and the switched fruit treatments (figure 4), whereas females fed the early-season fruit only suffered a delay in reproductive activity (time-to-event, *χ*^2^ = 15.017, d.f. = 2, *p* = 0.001; figure 4). Females fed the early-season fruit took 1.74 times longer, on average, to lay eggs than those females fed late-season fruit as adults (i.e. late-season fruit diet and switched fruit diet females). Additionally, late-season fruit females and switched fruit diet females laid between 38 and 44% of their total lifetime eggs in the first two weeks of reproduction. Females fed the early-season fruit only laid about 6% of their total lifetime eggs in that same timeframe.

## 4. Discussion

We used wild host plant diets to examine the effects that a seasonal shift in nutrition quality had on female reproductive success. We found that females that survived a poor early nutritional start were able to partially recover reproductive success when they were switched to higher quality adult diets. Females achieved partial recovery through laying eggs just as quickly as females fed a lifetime of late-season fruit, but interestingly females fed an improved adult diet could not produce as many eggs overall as the females fed a lifetime of late-season fruit. Further, females fed an improved diet at adulthood were unable to completely overcome the mortality consequences that poor early life nutrition inflicted. We also found that all fitness-related traits that we measured in this study were reduced for females fed early-season fruit compared with females fed late-season fruit. Taken together, our results suggest that females possess compensatory adaptations that help them overcome a poor early start by investing adult resources into reproductive recovery. However, we still found that a poor juvenile diet had lasting impacts on both survival and reproduction that an improved adult diet, even when ecologically relevant, could not fully overcome—supporting the silver spoon hypothesis.

One reason that *N. femorata* may not have fully reproductively recovered from poor early life nutrition is because of body space constraints. Arthropod external body size is fixed at adulthood due to their chitinous exoskeleton. External body sizes for females in this study differed by an average of 14.4%. Females fed early-season fruit had an external body size range from 2.87 to 4.27 mm with one outlier at 4.93 mm. Females fed late-season fruit had external body sizes that ranged from 3.52 to 5.01 mm (electronic supplementary material, S3). Body size and fecundity are highly correlated in insects [49,50] and external body size may restrict the amount of nutrition that they can acquire and/or place an upper limit on space for reproductive organs [51] resulting in lower lifetime egg production. For example, fecundity increases with body size in female water striders because larger females have relatively larger abdomens [52]. In most insects, ovaries are composed of several ovarioles that can each produce individual eggs [53]. These eggs are then stored in oviducts in the abdomen until females are ready to fertilize and lay them [53]. In *N. femorata* (LA Cirino 2018, unpublished data) and other insects [8,54–56], ovariole number is canalized. We found that the response to poor early food conditions in *N. femorata* is to develop slower and grow smaller bodies without altering ovariole number, which we found in a separate study (LA Cirino 2018, unpublished data). Thus, even if females were able to upregulate all ovarioles within each ovary once diet improved, their reproductive capacity may still be partially constrained by the smaller space they have to develop and store eggs within the body.

Juvenile diet had lasting effects on female survival and reproduction in this and other studies. The egg laying patterns we found are consistent with other existing research that used simulated natural [7] and novel shifts in diets [6,9]. However, the results of our study conflicted with some compensatory growth studies as an improved adult diet did not affect adult survival in these experiments [8,13]. A poor juvenile diet increased adult lifespan in zebra finches [13] but decreased adult survival in cockroaches [8] regardless of adult diet. These survival pattern differences might vary based on contrasting life-history strategies of these species. Yet, in Mevi-Schütz & Erhardt [7], an improved adult diet increased adult survival in map butterflies, which have a life history akin to cockroaches. These results are more consistent with those found in this study and in this case, as with our study, the simulated diet change is a scenario that map butterflies encounter in nature as seasons change. Conflicting results between this and the other studies that found no effect of adult diet on survivorship may be due to the seasonal change in host plant diet that *N. femorata*, like the map butterflies, encounter in nature, year after year. Since *N. femorata* females, like the map butterflies, experience these shifts yearly, it's possible that females were able to boost adult survival based on the evolutionary ties to their diet, an adaptation that females fed a novel shift in diet, like the cockroaches [8] and zebra finches [13], may not possess.

Animals may have evolved a ‘grow now, pay later’ mechanism of compensation if the fitness benefits of growth outweigh the costs [3]. Here, we show support for this compensatory adaptation to a poor early-nutritional start using the foods that our study organism feeds on in the wild and simulating a natural shift in resources that these animals experience annually. The benefits of allocating resources towards egg production should outweigh the survival costs in this species as long as females survive to reproductive age. Fitness in species that are primary consumers, relatively short lived and/or highly predated upon would probably be enhanced if compensation for poor early life conditions occurred by allocating resources towards reproduction at the cost of long-term survival. Compensatory mechanisms should evolve in this way because delays in reproduction could be devastating to female fitness in these species. By the time they reach sexual maturity, only a short reproductive window may be open for females to reproduce before mortality. However, females that belong to species that are long lived or are predators in a higher trophic level may not need to invest in quick reproduction. Rather, they may initially prioritize other life-history traits such as growth and/or survival. Examining species with contrasting life-history patterns under nutritional recovery would be a compelling avenue for future research. Finally, it is important to use the foods that organisms feed on in the wild in studies examining compensation, as we did in this study, so that they can provide us with more reliable life-history patterns that we can use to better understand the reproductive strategies and population dynamics of animals in nature.

## Data accessibility

Our data files used for analyses and R code are included as electronic files in the electronic supplementary material.
